# Optical Diagnostics in Human Diseases

**DOI:** 10.3390/diagnostics11050873

**Published:** 2021-05-12

**Authors:** Andrey Dunaev

**Affiliations:** Research and Development Center of Biomedical Photonics, Orel State University, 302026 Orel, Russia; dunaev@bmecenter.ru; Tel.: +7-4862-419806

**Keywords:** biophotonics, optics, spectroscopy, imaging, diagnostics

Light-based technologies provide unique opportunities for the diagnosis of various pathological disorders of biological tissues. With the advancement of modern science, they allow for the non-invasive identification of diseases at early stages. The optical technologies for obtaining information on the biochemical state and morphological structure of the investigated area are based on the assessment of light-tissue interaction (including laser radiation). For this purpose, a patient’s tissues and organs are probed with optical radiation, and reflected (scattered, passed through the tissue, re-emitted as fluorescence, etc.) light is recorded. The range of optical technologies applications in clinical practice is considerably wide since the optical properties of biological tissues are subject to significant changes in the course of the disease. Optical non-invasive diagnostics uses many spectroscopic and imaging techniques, including near infrared spectrophotometry, fluorescence spectroscopy (FS) and imaging, optical coherence tomography (OCT), confocal spectroscopy, optoacoustic tomography, laser Doppler flowmetry (LDF), laser speckle contrast imaging, and a number of other methods. However, despite the rapid development of optical methods in medical diagnostics, it should be borne in mind that most of them have not yet become the gold standard in clinical practice. They are mostly used in scientific research or as an additional clarifying method, increasingly using the so-called multimodal approach, where one diagnostic technology combines various optical and other physical research methods, which makes it possible to provide early diagnosis of functional changes before clinical manifestations of the disease based on the measurement results. In addition, the wider introduction of these methods into routine clinical practice is hindered by the insufficient elaboration of their methodological and technical support. This Special Issue of Diagnostics is devoted to the ideas regarding a solution to these problems.

The Special Issue highlights the challenges, advantages, and unique aspects of optical diagnostic methods for identifying and evaluating various diseases and pathological conditions. Articles focus on certain technologies, diseases, and various aspects of spectroscopy and imaging application in clinical practice.

Several studies in this Special Issue present new information relevant to surgical procedures, especially in oncology and gynecology. Zherebtsov and colleagues, according to the multimodal approach, described the development of the technical implementation and assessment of machine learning methods’ efficiency for the real-time diagnosis of tumors in hepatoduodenal organs by FS and LDF. This approach is aimed at improving the effectiveness of minimally invasive surgical operations by providing additional diagnostic information online [1]. Two articles are devoted to the topical problem of breast cancer’s early detection, including during surgery. Gubarkova and colleagues compared two types of optical coherence tomography–cross-polarization OCT (CP OCT) and a novel type of compressional optical coherence elastography. They confirmed the high potential of OCT-based examinations for rapid and accurate diagnostics during breast conservation surgery [2]. Iida and colleagues conducted Monte Carlo simulation of shortwave-infrared (SWIR) fluorescence photon migration in voxelized media for breast cancer detection. The obtained results showed that it is possible to predict the presence of early-stage breast cancer with high spatial resolution using SWIR and the phantom model in which the optical parameters are implemented in the breast structure [3]. Huang and colleagues proposed a promising method based on the gray level co-occurrence matrix (GLCM) image processing model to achieve a rapid technique with a more reliable diagnostic performance for various types of chemoresistance for the cisplatin of human ovarian adenocarcinoma cells by feature extraction of GLCM [4].

The problem of chronic or recurrent episodic pain in the urethra with unchanged urinalysis, the absence of any other clinical manifestations or somatically explainable causes, is complex and ultimately remains unresolved since the exact pathogenetic mechanisms are not yet fully understood. The article by Streltsova and colleagues is dedicated to the targeted study of female urethral tissues (their elasticity and the condition of the epithelial and connective tissue layers) in urethral pain syndrome (UPS). The results showed that the introduction of new CP OCT technology in conjunction with transvaginal compression ultrasound will allow for in vivo verification of structural changes in lower urinary tract tissues at their architectonics level and will provide more information about basic elements of the UPS pathogenesis [5].

Several studies in this Special Issue are devoted to otolaryngology and dentistry. Bryanskaya and colleagues developed a basis for instrument implementation of digital diaphanoscopy technology for the diagnosis of maxillary sinus inflammatory diseases, taking into account the anatomical, age, and gender features of patients. Their approach may be promising as a screening method for assessing the condition of maxillary sinuses both in hospitals and medical institutions, as well as remotely in the absence of otolaryngologists and diagnosticians [6].

Timchenko and colleagues studied the changes in tooth tissues in periodontitis using Raman spectroscopy for early and rapid diagnosis and the correction of treatment. The obtained results are a prerequisite for creating a device for rapid in vivo assessment of periodontitis based on changes in tooth enamel spectral values [7].

Savchenko and colleagues developed an experimental optical device for diagnosing liver diseases. The main advantages of the proposed device are its usability and fast presentation of results in real time. The device is based on optical densitometry. To determine the functional reserves of the liver, the researchers used indocyanine green. It is a non-toxic dye that binds well to blood proteins and is delivered to the liver through the bloodstream. The dye was administered intravenously to a patient, and then it was observed how long it took the liver to eliminate it from blood plasma. The concentration of indocyanine green in the blood was measured using the developed optical setup [8].

A number of articles are devoted to the study of alterations caused by diabetes mellitus (DM) and cardiovascular diseases. Kozlov and colleagues developed a new method of signal processing and data analysis in digital LDF. The main result of the study is the development of a set of classifiers that allow one to identify typical patterns of microcirculation in healthy volunteers and DM patients based on the presented diagnostic algorithm [9]. Fedorovich and colleagues studied the changes in microcirculation parameters in different body positions using a distributed system of wearable LDF devices. They demonstrated the significance of a body position influence during the monitoring of microcirculatory parameters. The results obtained may be of particular interest for further integration of an LDF channel into wearable devices for monitoring the state of cardiovascular systems [10]. Maslianitsyna and colleagues found the correspondence between in vivo and in vitro optical methods by studying the aggregation parameters in patients with cardiovascular and concomitant pathologies. Understanding the link between red blood cells (RBC) aggregation and widespread cardiovascular diseases is vital to creating new methods of diagnosis and treatment. In this work, the aggregation of RBC was studied using different optical in vivo and in vitro measurement techniques. In vivo and in vitro methods yielded correlated results: the faster the cells moved in the capillaries, the less cells aggregated in vitro. DM had an additional significant effect on the aggregation properties of coronary heart disease patients. These findings are prominent for diagnosing and monitoring the state of patients with pathologies that affect blood properties [11]. Machikhin and colleagues reported on an in vivo stain-free blood vessel imaging technique for the analysis of zebrafish embryonic development. The developed algorithm for processing bright-field microscopy images enables detection, mapping, and quantitative characterization of cardiac activity across the whole embryo. To validate the proposed approach, the blood flow velocity and heart rate dynamics were evaluated for multiple embryos at pre-larval stages. This non-invasive technique may shed light on the mechanism of vessels’ activity initiation as well as the cardiovascular system resistance to environmental stresses [12].

Finally, Batool and colleagues reviewed and compiled the optical properties of human tissues and the circulatory system, especially blood. One of the main conclusions of this review is that there are numerous physical and methodological factors important for optical properties research and that one should be aware of them before performing their own measurements. The authors pointed out the main factors that affect absorption spectra of whole blood and hence influence optical properties. Revision of available polarimetric techniques can be helpful for readers who practice biomedical optics methods [13].

Thus, the presented Special Issue reflects novel innovative research and emerging ideas in optical non-invasive diagnostics for their wider translation into clinical practice, e.g., for the development of wearable technologies, personalized medicine, and robotic surgery.

## Data Availability

Not applicable.
